# Anterior sternoclavicular joint disruption with ipsilateral medial clavicle fracture in an adolescent: case report and literature review

**DOI:** 10.1016/j.xrrt.2026.100697

**Published:** 2026-02-20

**Authors:** Ahmed Alabdali, Omar AlQattan, Hanan Ahmad, Malak Almutairi, Abdullah Al Jubori

**Affiliations:** a Orthopedic Department, Dr Soliman Fakeeh Hospital, Jeddah, Saudi Arabia; b Faculty of Medicine, King Abdulaziz University, Jeddah, Saudi Arabia

**Keywords:** Sternoclavicular joint, Clavicle fracture, Physeal injury, Adolescent trauma, Open reduction, Case report

## Introduction

Dislocation of the sternoclavicular joint (SCJ) is a rare pathology in orthopedic trauma, with some studies reporting it accounts for less than 5% of all shoulder girdle injuries.^18^ In adults, these injuries typically occur as true dislocations following capsular and ligamentous disruption, whereas in skeletally immature patients whose medial clavicular physis is the last in the body to fuse, usually between 22 and 25 years of age, they often present as physeal fracture-dislocations or “pseudodislocations.” The anterior dislocation may result in ongoing pain and discomfort with some loss of function, while posterior dislocation carries far more serious potential complications due to possible injury to mediastinal structures.^4^^,^^15^^,^^21^ The incidence of physeal fractures of the medial clavicle is not well established, but this site is considered one of the rarest locations for physeal injury described in the literature.^4^ We report a rare case of anterior SCJ disruption with an associated ipsilateral clavicle fracture. Our goal with this report is to expand on the very limited data on SCJ disruption and ipsilateral clavicle fracture, thereby helping guide recognition and management in similar cases.

## Case report

### Initial assessment

A 15-year-old male presented initially to an outside hospital after he was involved in a high-speed road traffic accident with vehicle rollover. He was hemodynamically stable, alert, and oriented. He reported isolated pain localized to the left shoulder, without respiratory distress, dysphagia, neurological deficit, or signs of vascular compromise.

**Figure 1:**
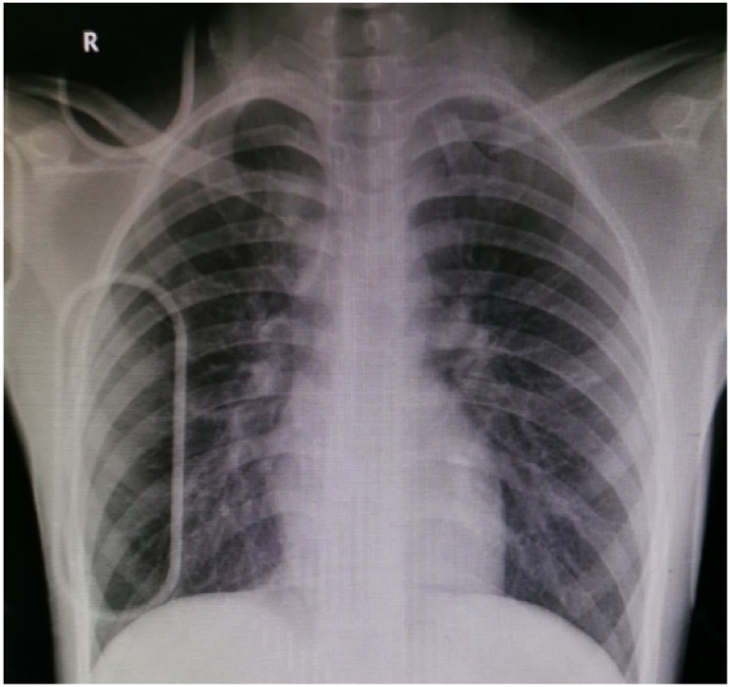
Initial anteroposterior chest radiograph demonstrating medial clavicle fracture with associated sternoclavicular joint disruption on the left side.

**Figure 2:**
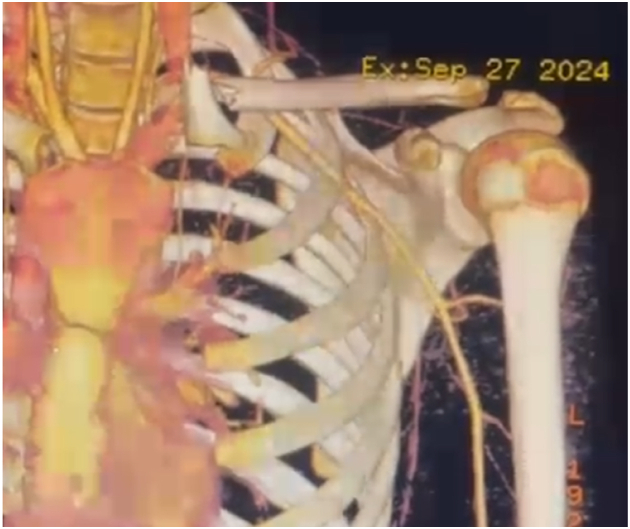
Three-dimensional computed tomography (CT) angiographic reconstruction showing anterior dislocation of the left sternoclavicular joint and 90° rotation of the medial clavicle fragment without evidence of vascular compression.

Plain radiographs demonstrated a medial clavicle fracture with associated SCJ disruption (Fig. 1). Computed tomography (CT) angiography of the SCJs confirmed anterior dislocation of the left SCJ with an associated medial clavicle fracture. The medial fragment was free-floating, rotated approximately 90° in the coronal plane, with its lateral aspect pointing superiorly and the sternal aspect directed inferiorly and anteriorly (Fig. 2). There was no evidence of compression of the great vessels.

The patient was referred to our institution for definitive management. On examination, he was neurovascularly intact with an obvious prominence and deformity over the left SCJ and overlying ecchymosis. No attempt at closed reduction had been made at the referring hospital, and no magnetic resonance imaging (MRI) was performed.

### Decision-making

The decision for operative fixation of this injury pattern was made based on the severe displacement of the fracture and the apparent instability of the free-floating fragment. Consideration of a fixation construct spanning both the medial clavicle fracture and the SCJ was done, but ultimately decided against due to excessive soft tissue stripping needed for the spanning construct and the absolute need for future hardware removal of fixation spanning SCJ. The final pre-operative plan was for standard plating of the medial clavicle fracture and intraoperative assessment for any need for further fixation of the SCJ. The patient was booked and underwent the surgery 7 days after the initial injury.

### Operative technique

Definitive management was performed with open reduction and internal fixation of the left medial clavicle fracture. The patient is in the beach chair position. The left clavicular region was prepped and draped in the usual sterile fashion. A cardiothoracic surgeon was on standby. A skin incision was made over the superior medial clavicle, followed by meticulous subcutaneous dissection, with identification and protection of the medial branch of the supraclavicular nerve.

**Figure 3:**
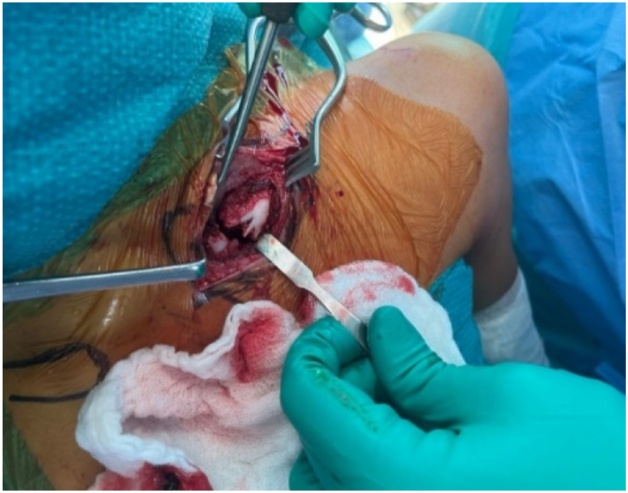
Intraoperative exposure of the left medial clavicle showing the rotated free-floating fragment prior to reduction.

**Figure 4:**
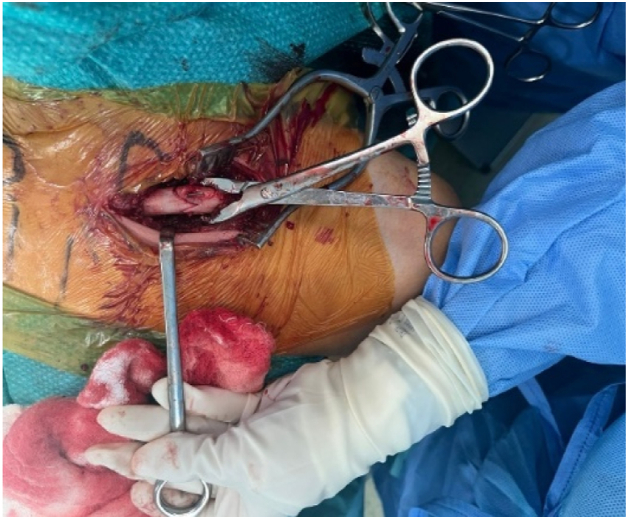
Anatomical reduction achieved and provisionally secured with a K-wire under fluoroscopic guidance.

The medial clavicle fracture was visualized, showing 90° rotation of the fragment consistent with CT findings (Fig. 3). Exposure of the clavicle was limited to only what was deemed necessary for the reduction of the clavicle fracture and for the placement of the plate; no attempt was made to expose the SCJ. Anatomical reduction was achieved with bone clamps and temporarily secured with a provisional K-wire (Fig. 4). Reduction of the clavicle fracture restored normal alignment of the SCJ on intraoperative fluoroscopy, showing reduction of the rotational element of the SCJ disruption on anterior–posterior views.

**Figure 5:**
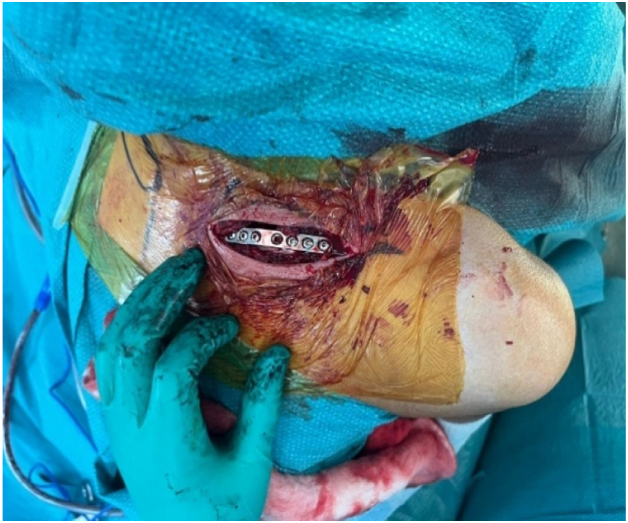
Definitive fixation using a superiorly placed 2.7 mm reconstruction plate secured with 3 bicortical screws on each side of the fracture.

**Figure 6:**
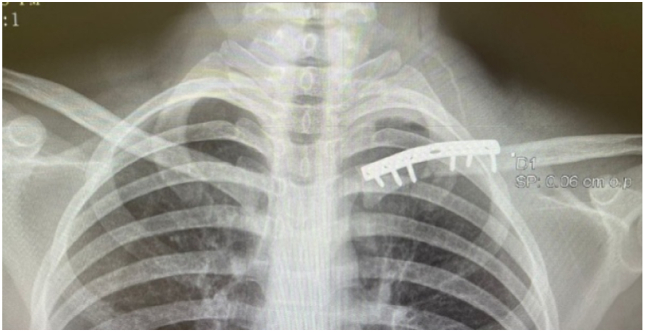
Post-operative anteroposterior radiograph demonstrating satisfactory hardware placement and restoration of sternoclavicular joint alignment.

Definitive fixation was achieved with a superiorly placed 2.7 mm reconstruction plate, secured with 3 bicortical screws on each side of the fracture (Fig. 5). No additional fixation or suturing of any ligaments was done to the SCJ. Wound closure was in standard layered fashion of fascia over clavicle, platysma, subcutaneous tissue, and skin. Final intraoperative imaging confirmed satisfactory hardware placement and reduction of both the fracture and the SCJ (Fig. 6).

### Post-operative course and follow-up

Post-operatively, the patient's arm was immobilized in a sling for one week, followed by a gradual return to active range of motion as tolerated. At the 1-week follow-up, forward flexion was 90°. By the 6-week review, the patient achieved forward flexion to 160°, abduction to 150°, and full functional use without pain. At 3 months, he had a full and symmetrical range of motion of the left shoulder compared with the contralateral side, with no tenderness over the SCJ and no radiographic evidence of loss of reduction or hardware complications. At the final follow-up (6 months), he remained asymptomatic, had returned to unrestricted activities, and radiographs confirmed complete union of the fracture with maintained joint congruity.

## Discussion

### Causes and mechanism of injuries

The most common cause of SCJ injury is road traffic accidents, followed by sports-related trauma. The mechanism is often classified into direct and indirect forces. Indirect forces are applied to the shoulder and transmitted to the SCJ.^16^ Combined injuries usually result from an amalgamation of multiple forces, as a single force would likely dissipate after causing one injury. This was the case in our patient, who sustained a rollover road traffic accident.

### Dislocation vs. physeal injury

The medial clavicular physis does not begin ossification until approximately 18 years of age and does not fuse till around 25 years of age. SCJ dislocations in skeletally immature patients may not be a true dislocation but a physeal fracture sometimes termed pseudodislocation.^10^ This distinction is clinically significant. In true dislocations, there is disruption of the SCJ capsule and often associated injury to the costoclavicular and interclavicular ligaments, depending on whether the displacement is anterior or posterior.^7^ In contrast, physeal injuries may preserve these ligamentous attachments, as the periosteum and attached ligaments remain intact and avulse with the metaphyseal fragment.^4^ In skeletally immature patients with physeal injuries, a substantial degree of remodeling can be expected, which may make nonoperative management (“skillful neglect”) a viable option in certain anterior injuries. However, posterior injuries whether true dislocations or displaced physeal fractures carry the risk of serious mediastinal complications and often require urgent reduction.^15^ There are some rare reports of misdiagnosed chronic dislocation later being found to be pseudodislocation nonunions.^5^

### Imaging

Due to the anatomical location of the SCJ, it is difficult to accurately assess the direction of injury on standard radiographs, and CT scanning is often required. CT angiography provides the added advantage of evaluating for associated vascular compromise. MRI is valuable for assessing ligamentous integrity and for differentiating true dislocations from pseudodislocations.^10^ Some surgeons do not routinely perform MRI, as the definitive nature of the injury can often be determined intraoperatively.^11^

### Treatment

Because of the rarity of this specific injury pattern, the available evidence regarding its management is limited to case reports.

### Closed reduction

Closed reduction of isolated physeal injuries can be successful, particularly if performed within 48 hours. It should be considered in all isolated physeal injuries, especially in posterior injuries, where there have been reports of severe complications such as pneumothorax, injury to the great vessels, tracheal injury, esophageal compression leading to dysphagia, and death.^18^ However, closed reduction maneuvers are described primarily for isolated SCJ injuries, not for combined injuries such as in our case, where a free-floating fragment was present. In such cases, there is a theoretical risk that attempted closed reduction could displace the fragment posteriorly, potentially resulting in catastrophic complications. There is a report of an ipsilateral SCJ dislocation with clavicle fracture in which closed reduction was attempted and failed, but no serious complication occurred.^19^

### Operative fixation

A meta-analysis on SCJ dislocation identified irreducible posterior SCJ dislocation as an indication for open surgical treatment, while irreducible anterior dislocation in a healthy, active patient was considered a relative indication, based on clinical outcomes.^17^ Another meta-analysis focusing solely on posterior SCJ injuries in the adolescent population aimed to compare the outcomes of open and closed treatment methods. However, due to limited follow-up and significant variability in the techniques employed, the authors concluded that a formal statistical comparison was not possible.^18^

Regarding operative indications for isolated clavicle fractures in adolescents, a recent review states that the only absolute indications for surgery are open fractures, fractures with skin tenting, and major venous or arterial injury. Most other injuries are managed conservatively.^13^

### Literature review

Review of the literature for ipsilateral SCJ disruption, whether in the adult or pediatric population, with associated clavicle fracture yielded a few results. Various treatment methods were implemented with generally good results (Table I).

**Table I:** Literature review of ipsilateral sternoclavicular joint disruption or dislocation with clavicle fracture.

Title (Yr)	Authors	The injuries	Imaging	Open vs. closed reduction	Fixation method	Follow-up	Complications
Sternoclavicular physeal fracture. (2008)^11^	Lampasi et al.	14-yo boy; medial clavicle physeal fracture + adjacent clavicle fracture	Radiographs + 3D CT	Open reduction (no closed attempt reported)	Clavicle plating (reconstruction plate) + periosteal suture	7 mo; full ROM, returned to sports	None reported
Anterior SCJ dislocation with clavicle fracture (2013)^9^	Khalid et al.	17-yo male; anterior SCJ dislocation + mid-shaft clavicle fracture	X-ray/CT	Open reduction	Clavicle plating + SCJ fusion	Healed, but limited ROM compared to contralateral	Limited ROM noted
Fracture and retrosternal SC dislocation (1978)^8^	Kanoksikarin and Wearne	19-yo male; clavicle fracture + posterior SCJ dislocation	Not specified	Open reduction	Pin fixation + SCJ capsular repair	17 mo; full ROM, returned to sports	None noted
SCJ disruption + clavicle fracture (2010)^20^	Tompkins et al.	9-yo boy; SCJ disruption + clavicle fracture	X-ray/CT	Open reduction	Fiber-Wire transosseous suture	6 mo; full ROM, returned to sports	None noted
Epiphyseal separation + clavicle fracture (1984)^12^	Lemire and Rosman	15-yo boy; epiphyseal separation + adjacent clavicle fracture	X-ray/CT	Open reduction	Figure-of-8 sutures through lateral fragment + periosteal suturing	1 yr; returned to normal activity	Not reported
Hook-plate fixation case series (2022)^23^	Zhang et al.	17 patients >16 yo; proximal clavicle fracture + SCJ dislocation	X-ray + CT	Open reduction (hook-plate applied)	Hook-plate into sternum + screws into clavicle; in posterior cases a spacer/cap is added at the hook end	Not reported; assumed union	Two patients had complications (internal fixation failure; fracture nonunion)
Medially displaced fracture-dislocation (2023)^1^	Afifi et al.	18-yo male; medial clavicle fracture + posterior SCJ disruption	CT (rotated fragment noted)	Open reduction	Clavicle plating + figure-of-8 tape arthrodesis	5 mo; healed, returned to training	None reported
Anterior SCJ dislocation + clavicle fracture (2021)^14^	Reuter et al.	45-yo male; clavicle fracture + anterior SCJ dislocation	X-ray + CT	Open reduction	Transosseous SCJ suture + clavicle locking plate	12 weeks; healed, returned to pre-injury activity	None reported
Transosseous + plating in teens (2018)^3^	Barrera-Ochoa et al.	13- and 15-yo; medial clavicle fracture + posterior SCJ dislocation	X-ray + CT	Open reduction	Transosseous SCJ suture + clavicle plate	First patient at 24 mo, second at 13 mo: both full ROM	None noted
SCJ suture only (female, age 50) (2019)^22^	Yadav et al.	50-yo female; SCJ dislocation only (clavicle fracture implicit but not fixed)	X-ray + CT	Open reduction	Transosseous SCJ suture only	6 mo; fracture healed, SCJ stable, full ROM	None reported
Additional reports (1992; 1999)^6^	Hardy; Allen and Zielinski	Various ages with combined injuries	Not specified	Likely open	Varies	Follow-up varies	-
Meta-analysis cautioning on K-wire use (2014)^2^	Allen Jr B	Warning about pin/K-wire complications	—	—	—	—	-

*SCJ*, sternoclavicular joint; *ROM*, range of motion; *3D*, three-dimensional; *CT*, computed tomography; *yo*, year-old.

It should be noted many authors are against pin or K-wire fixation in SCJ dislocations due to the possible complication of pin migration that has led to death in some cases.^18^

## Conclusion

Ipsilateral SCJ disruption with clavicle fracture is rare, particularly in adolescents. Our case of a 15-year-old male treated with open reduction and rigid fixation of the medial clavicle using a reconstruction plate achieved stable SCJ alignment without direct joint fixation and resulted in full functional recovery. This case adds to the limited literature and supports the role of rigid clavicular fixation as a safe and effective treatment option in similar presentations.

## Disclaimers:

Funding: No funding was disclosed by the authors.

Conflicts of interest: The authors, their immediate families, and any research foundations with which they are affiliated have not received any financial payments or other benefits from any commercial entity related to the subject of this article.

Patient consent: Obtained.
